# Supplementary material for: Early Strokes Are Associated with More Global Cognitive Deficits in Adults with Sickle Cell Disease
Source: J Clin Med. 2023 Feb 17;12(4):1615. doi: 10.3390/jcm12041615 (PMC9967394; doi:10.3390/jcm12041615)

Supplemental Figure S1. Initial eigenvalue and screeplot of the principal component analysis of cognitive tests

Initial Eigenvalues

Component	Eigenvalue	% of Variance	Cumulative %
1	7.8667	41.40392	41.4
2	1.8584	9.78124	51.2
3	1.5278	8.04102	59.2
4	1.3627	7.17234	66.4
5	1.0648	5.60434	72.0
6	0.9095	4.78699	76.8
7	0.7698	4.05148	80.8
8	0.6935	3.65003	84.5
9	0.5991	3.15307	87.6
10	0.4869	2.56260	90.2
11	0.4533	2.38592	92.6
12	0.3456	1.81907	94.4
13	0.2893	1.52253	95.9
14	0.2609	1.37297	97.3
15	0.2243	1.18070	98.5
16	0.1527	0.80347	99.3
17	0.0998	0.52536	99.8
18	0.0339	0.17819	100.0
19	9.07e-4	0.00477	100.0

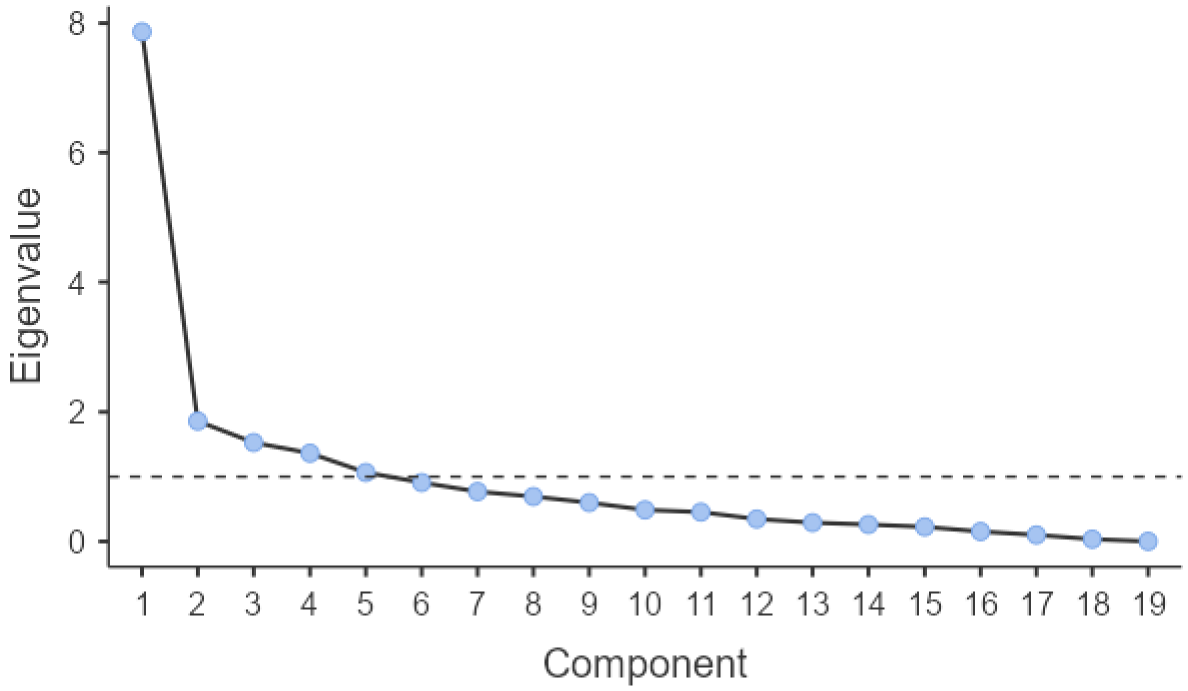

Supplement: Supplementary file 1 [file jcm-12-01615-s001.zip › jcm-2172853-supplementary.pdf]
